# Supplementary material for: Dpp controls growth and patterning in Drosophila wing precursors through distinct modes of action
Source: eLife. 2017 Jul 4;6:e22546. doi: 10.7554/eLife.22546 (PMC5560859; doi:10.7554/eLife.22546)
Supplement: Source data 1. — DOI: http://dx.doi.org/10.7554/eLife.22546.019 [file elife-22546-data1.docx]

E datDNA sequence for fragment re-inserted in the attP site of *dpp*KO mutant to generate *dpp^FRT-CA^*

gcggccgcTAGAGGTAGTCCTTTTTTTTAGATTCAATAGCTAAGTTcctatgatttctttgctccaagcTCACCCGCAAT

ATCCTTCTTTTCCGTTTCCTTGCAGATagtaaaatcgacgatcgatttccgcaccaccaccggtttcggctgcacttcga

cgtgaagagcattcccgccgacgagaagctgaaggcggcggagCTGCAGctgaCCCGGGacgcactcagtcaacaggtgg

tggccagcagatcgtcggcgaatcggacgcgctaccaggtgcttgtctacgacatcacgcgcgtcggggtgcgtggtcag

cgggagccgagctatctgctgttggacaccaagacggtccggcttaacagcacggacacggtgagcctcgatgtccagcc

ggccgtggaccggtggctggcgagtccgcagcgcaactacggactgctggtggaggtgcggacggtccgctccctgaagc

cggccccacaccaccatgtacgcctgcgccgcagcgcggaCGAGGCGCACGAGCGGTGGCAGCacaagcagccGCTCCTG

TTCACCTACACGGACGacgggcggcacaaggcgcgctccattcgggacgtgtctggcggagagggcggtggcAAGGGCGG

CCGGAACAAGCGGCAGCCGAGACGGCCTACGAGGCGCAAGAACCACGACGACtacccatacGACGTCcctgactatgcgG

GCGGAtatccctatgatgttccagattacgctacctgccggcggcACTCGCTGTACGTGGACTTCTCGGACGTGGGCTGG

GACGACTGGATTGTggcgccTCTGGGCTACGATGCATATTACTGCCACGGGAAGTGCCCCTTCCCGCTGGCCGACCACTT

TAACTcgaccaatcacgccgtggtgcagACCCTGGTCAACAATATGAATCCCggcaaggtgccgaaggcgtgctgCGTGC

CCACGCAACTGGACAGCGTGGCCATGCTCTATCTCAACGACCAAAGTACGGTGGTGCTGAAGAACTACCAGGAGATGACC

GTGGTGGGCTGTGGCTGTCGATAGATTCGCACCACCATCGCACTAGTgACcataccacgccatccactcaacCGAGTGAA

TGCGATGGGAAATCGCGAGCGAGAGAGCATCAAATGCTGTTTGGTTCCAAGCCGTCAATGCTTTAAACACAACGCAAACA

AAATGGACTGAATATTTGAATTTTAAGTGTAAATCGTTAGACTTTAGCCGTATCGAGTAACGAGCAAACAGGCGGCAGCC

ACGCCCACATCCACGTCCCCACCAAAACCGCCCGCCTTGGAGCCTCTGTCGATTTCCCCAGCCAGGCTGGCGAAAAATCC

CAGATCAGAGTGCAGATTTGAGAGCGCAGAGTCCACTGTATATAGCCGCCATGCCACGCCCCCAACACAGATAGTCCCCG

CCCATCCGCCAGATACTTCAGATATTAGATACTTTCGTATCTGTGTGCGCTGCTGCTGCTGAAGGAGAAGTTAAGGGAGG

AAAAGAGGAGTATGCTTAGGAGTAAGAGCGACCAATTGAACAAATTGTATAGAAATGCTAATATATATTAAAAAACCCTA

TCGATGCGAACTGGTATCTTTGTATACATTTGTACATGTATGTGGAAAGGAGACCTATTCTACTAGCCGTTTTTGTTAAT

AATTTTATAAAGCAATAGCAAACCACTTGTAAATTAACTAGCGAGAGCATAACCGAATAATGACTTGAAATTACTTAGGA

ACTATCATCCTAAACACATAGTTGTAGAAAGACCAGAAAAACAAACAGATATTGCATATGTAACTCTCTTGTATATGTAC

TAAACACCTATATACTTTATATGCGGTACACACTCACTCACCCCCATTAGCAAACACACAACCACACACACATATCGACG

AAAGGGTATTCAAACTTCGTTGCGCATTCAACTAAACGTAACTGTATAAACAAAACGAATGCCCTATAAATATATGAATA

ACTATCTACATCGTTATGCGTTCTAAGCTAAGCTCGAATAAATCCGTAAACGTTAATTAATCTAGAATCGTAAGACCTAA

CGCGTAAGCTCAGCATGTTGGATAAATTAATAGAAACGAGAGAAAAGAGAAAAAACCCCACAAAAAGAAAACCCGATAAA

TGGAAAATATCGATTCGTGCCTGATGTTGCAGCGCACGTCTCGTATATGCAGTTTGTCATATAAACATTATTATTTTATT

TATTTAAAACAACCCGTATTTTTGAGGACGACGACGATGATGCAGGAGCAAGGATGAAAAGAAAGATGAAAAATATAAAA

GAAAACAATTTATTAAAAAAAAAATATATATACAATGGTCTTTATTTACTACGGATTACTGGTGAGGGATAAAAGAAAAG

TATATGGTATACATATATATGGAAAAAAAAGTTATCCTTCGAGCCGGATTTGAACCAGCGACCTATGGATGCCTGCTGGA

TTCGATCACCTGGAAAGTGTGATCAGATCTGAAAACTTCTACAGTCCACCGCTCTACCAACTGAGCTATCGAAGGTGATA

GCAGCTCAGGTGTCAATTTAAAATTTCGCCAGCTTATCAAAATTAGTTCAAGTACTCGGCAAAAAGCAGATTCAAGTGAA

ATAATGGCAAAAAGCTTATCGCAGCATTAAAACGCAGCAACAACACCGTCACAGGTATACCCCAATACGTGTTGATAAGA

GAGAGggaagcaagtgccaaagcgcctcgagtGGCGCGCC

DNA sequence for fragment re-inserted in the attP site of *dpp^KO-PSB^* mutant to generate *dpp^FRT-PSB^*

CAATTCATTACCGTACGACGAAGTTCCTATTCCGAAGTTCCTATTCTCTAGAAAGTATAGGAACTTCGAATTCCAAGCGATACCACTCGACCATAGGAAACTTTATAAGACTGAGAGTTGCAAGCGACCATGCGCGCATGGCTTCTACTCCTCGCAGTGCTGGCGACTTTTCAAACGATTGTTCGAGTTGCTAGCACCGAGGATATATCCCAGAGATTCATCGCCGCCATAGCGCCCGTTGCCGCTCATATTCCGCTAGCATCAGCATCAGGATCAGGATCAGGACGATCTGGATCTAGATCGGTAGGAGCCTCGACCAGCACAGCATTAGCAAAAGCATTTAATCCATTCAGCGAGCCCGCCTCGTTCAGTGATAGTGATAAAAGCCATCGGAGTAAAACAAACAAAAAACCTAGCAAAAGTGACGCGAACCGACAGTTCAACGAAGTGCATAAGCCAAGAACAGACCAATTAGAAAATTCCAAAAATAAGTCTAAACAATTAGTTAATAAACCCAACCACAACAAAATGGCTGTCAAGGAGCAGAGGAGCCACCACAAGAAGAGCCACCACCATCGCAGCCACCAGCCAAAGCAGGCCAGTGCATCCACAGAATCTCATCAATCCTCGTCGATTGAATCAATCTTCGTGGAGGAGCCGACGCTGGTGCTCGACCGCGAGGTGGCCTCCATCAACGTGCCCGCCAACGCCAAGGCCATCATCGCCGAGCAGGGCCCGTCCACCTACAGCAAGGAGGCGCTCATCAAGGACAAGCTGAAGCCAGACCCCTCCACTCTAGTCGAGATCGAGAAGAGCCTGCTCTCGCTGTTCAACATGAAGCGCCCGCCCAAGATCGACCGCTCCAAGATCATCATCCCCGAGCCGATGAAGAAGCTCTACGCCGAGATCATGGGCCACGAGCTCGACTCGGTCAACATCCCCAAGCCGGGTCTGCTGACCAAGTCGGCCAACACAGTGCGAAGTTTTACACACAAAGATAGTAAAATCGACGATCGATTTCCGCACCACCATCGGTTTCGGCTGCACTTCGACGTGAAGAGCATTCCCGCCGACGAGAAGCTGAAGGCGGCGGAGCTGCAGCTGACCCGGGACGCACTCAGTCAACAGGTGGTGGCCAGCAGATCGTCGGCGAATCGGACGCGCTACCAGGTGCTTGTCTACGACATCACGCGCGTCGGGGTGCGTGGTCAGCGGGAGCCGAGCTATCTGCTGTTGGACACCAAGACGGTCCGGCTTAACAGCACGGACACGGTGAGCCTCGATGTCCAGCCGGCCGTGGACCGGTGGCTGGCGAGTCCGCAGCGCAACTACGGACTGCTGGTGGAGGTGCGGACGGTCCGCTCCCTGAAGCCGGCCCCACACCACCATGTACGCCTGCGCCGCAGCGCGGACGAGGCGCACGAGCGGTGGCAGCACAAGCAGCCGCTCCTGTTCACCTACACGGACGACGGGCGGCACAAGGCGCGCTCCATTCGGGACGTGTCTGGCGGAGAGGGCGGTGGCAAGGGCGGCCGGAACAAGCGGCAGCCGAGACGGCCTACGAGGCGCAAGAACCACGACGACGCGGCCGCCTACCCGTACGACGTGCCCGATTATGCCGGCTACCCCTACGATGTGCCGGACTACGCCGGCTCCTACCCCTATGACGTGCCCGATTACGCCGCGGCCGCCACCTGCCGGCGGCACTCGCTGTACGTGGACTTCTCGGACGTGGGCTGGGACGACTGGATTGTGGCGCCTCTGGGCTACGATGCATATTACTGCCACGGGAAGTGCCCCTTCCCGCTGGCCGACCACTTTAACTCGACCAATCACGCCGTGGTGCAGACCCTGGTCAACAATATGAATCCCGGCAAGGTGCCGAAGGCGTGCTGCGTGCCCACGCAACTGGACAGCGTGGCCATGCTCTATCTCAACGACCAAAGTACGGTGGTGCTGAAGAACTACCAGGAGATGACCGTGGTGGGCTGTGGCTGTCGATAGATTCGCACCACCATCGCACCATACCACGCCATCCACTCAACCGAGTGGATGCGATGGGAAATCGCGAGCGAGAGAGCATCAAATGCTGTTTGGTTCCAAGCCGTCAATGCTTTAAACACAACGCAAACAAAATGGACTGAATATTTGAATTTTAAGTGTAAATCGTTAGACTTTAGCCGTATCGAGTAACGAGCAACAGGCGGCAGCCACGCCCACATCCACGTCCCCACCAAAACCGCCCGCCTTGGAGCCTCTGTCGATTTCCCCAGCCAGGCTGGCGAAAAATCCCAGATCAGAGTGCAGATTTGAGAGCGCAGAGTCCACTGTATATAGCCGCCATGCCACGCCCCCAACACAGATAGTCCCCGCCCATCCGCCAGATACTTCAGATATTAGATACTTTCGTATCTGTGTGCGCTGCTGCTGCTGAAGGAGAAGTTAAGGGAGAAAAAGAGGAGTATGCTTAGGAGTAAGAGCGACCAATTGAACAAATTGTATAGAAATGCTAATATATATTAAAAAACCCTATCGATGCGAACTGGTATCTTTGTATGTACATGTATGTGGAAAGGAGACCTATTCTACTAGCCGTTTTTGTTAATAATTTTATAAAGCAATAGCAAACCACTTGTAAATTAACTAGCGAGAGCATAACCGAATAATGACTTGAAATTACTTAGGAACTATCATCCTAAACACATAGTTGTAGAAAGACCAGAAAAACAAACAGATATTGCATATGTAACTCTCTTGTATATGTACTAAACACCTATATACTTTATATGCGGTACACACTCACTCACCCCCATTAGCAAACACACAACCACACACACACACATATCGACGAAAGGGTATTCAAACTTCGTTGCGCATTCAACTAAACGTAACTGTATAAACAAAACGTATGCCCTATAAATATATGAATAACTATCTACATCGTTATGCGTTCTAAGCTAAGCTCGAATAAATCCGTAAACGTTAATTAATCTAGAATCGTAAGACCTAACGCGTAAGCTCAGCATGTTGGATAAATTAATAGAAACGAGAGAAAAGAGAAAAAACCCCACAAAAAGAAAACCCGATAAATGGAAAATATCGATTCGTGCCTGATGTTGCAGCGCACGTCTCGTATATGCAGTTTGTCATATAAACATTATTATTTTATTTATTTAAAACAACCCGTATTTTTGAGGACGACGACGATGATGCAGGAGCAAGGATGAAAAGAAAGATGAAAAATATAAAAGAAAACAATTTATTAAAAAAAAAATATATATACAATGGTCTTTATTTACTACGGATTACTGGTGAGGGATAAAAGAAAAGTATATGGTATACATATATATGGAAAAAAAAGTTATCCTTCGAGCCGGATTTGAACCAGCGACCTAGCTAGGATCCA

DNA sequence for fragment re-inserted in the attP site of *dpp*KO mutant to generate *dpp^FRT-REP^*

gcggccgcTAGAGGTAGTCCTTTTTTTTAGATTCAATAGCTAAGTTcctatgatttctttgctccaagcTCACCCGCAAT

ATCCTTCTTTTCCGTTTCCTTGCAGATagtaaaatcgacgatcgatttccgcaccaccaccggtttcggctgcacttcga

cgtgaagagcattcccgccgacgagaagctgaaggcggcggagCTGCAGctgaCCCGGGacgcactcagtcaacaggtgg

tggccagcagatcgtcggcgaatcggacgcgctaccaggtgcttgtctacgacatcacgcgcgtcggggtgcgtggtcag

cgggagccgagctatctgctgttggacaccaagacggtccggcttaacagcacggacacggtgagcctcgatgtccagcc

ggccgtggaccggtggctggcgagtccgcagcgcaactacggactgctggtggaggtgcggacggtccgctccctgaagc

cggccccacaccaccatgtacgcctgcgccgcagcgcggaCGAGGCGCACGAGCGGTGGCAGCacaagcagccGCTCCTG

TTCACCTACACGGACGacgggcggcacaaggcgcgctccattcgggacgtgtctggcggagagggcggtggcAAGGGCGG

CCGGAACAAGCGGCAGCCGAGACGGCCTACGAGGCGCAAGAACCACGACGACtacccatacGACGTCcctgactatgcgG

GCGGAtatccctatgatgttccagattacgctacctgccggcggcACTCGCTGTACGTGGACTTCTCGGACGTGGGCTGG

GACGACTGGATTGTggcgccTCTGGGCTACGATGCATATTACTGCCACGGGAAGTGCCCCTTCCCGCTGGCCGACCACTT

TAACTcgaccaatcacgccgtggtgcagACCCTGGTCAACAATATGAATCCCggcaaggtgccgaaggcgtgctgCGTGC

CCACGCAACTGGACAGCGTGGCCATGCTCTATCTCAACGACCAAAGTACGGTGGTGCTGAAGAACTACCAGGAGATGACC

GTGGTGGGCTGTGGCTGTCGATAGATTCGCACCACCATCGCACTAGTgACcataccacgccatccactcaacCGAGTGAA

TGCGAtgggaaatcgcgagcgagagagcATCAAATGCTGTTTGGTTCCAAGCCGTCAATGCTTTAAACACAACGCAAACA

AAATGGACTGAATATTTGAATTTTAAGTGTAAATCGTTAGACTTTAGCCGTATCGAGTAACGAGCAAACAGGCGGCAGCC

ACGCCCACATCCACGTCCCCACCAAAACCGCCCGCCTTGGAGCCTCTGTCGATTTCCCCAGCCAGGCTGGCGAAAAATCC

CAGATCAGAGTGCAGATTTGAGAGCGCAGAGTCCACTGTATATAGCCGCCATGCCACGCCCCCAACACAGATAGTCCCCG

CCCATCCGCCAGATACTTCAGATATTAGATACTTTCGTATCTGTGTGCGCTGCTGCTGCTGAAGGAGAAGTTAAGGGAGG

AAAAGAGGAGTATGCTTAGGAGTAAGAGCGACCAATTGAACAAATTGTATAGAAATGCTAATATATATTAAAAAACCCTA

TCGATGCGAACTGGTATCTTTGTATACATTTGTACATGTATGTGGAAAGGAGACCTATTCTACTAGCCGTTTTTGTTAAT

AATTTTATAAAGCAATAGCAAACCACTTGTAAATTAACTAGCGAGAGCATAACCGAATAATGACTTGAAATTACTTAGGA

ACTATCATCCTAAACACATAGTTGTAGAAAGACCAGAAAAACAAACAGATATTGCATATGTAACTCTCTTGTATATGTAC

TAAACACCTATATACTTTATATGCGGTACACACTCACTCACCCCCATTAGCAAACACACAACCACACACACATATCGACG

AAAGGGTATTCAAACTTCGTTGCGCATTCAACTAAACGTAACTGTATAAACAAAACGAATGCCCTATAAATATATGAATA

ACTATCTACATCGTTATGCGTTCTAAGCTAAGCTCGAATAAATCCGTAAACGTTAATTAATCTAGAATCGTAAGACCTAA

CGCGTAAGCTCAGCATGTTGGATAAATTAATAGAAACGAGAGAAAAGAGAAAAAACCCCACAAAAAGAAAACCCGATAAA

TGGAAAATATCGATTCGTGCCTGATGTTGCAGCGCACGTCTCGTATATGCAGTTTGTCATATAAACATTATTATTTTATT

TATTTAAAACAACCCGTATTTTTGAGGACGACGACGATGATGCAGGAGCAAGGATGAAAAGAAAGATGAAAAATATAAAA

GAAAACAATTTATTAAAAAAAAAATATATATACAATGGTCTTTATTTACTACGGATTACTGGTGAGGGATAAAAGAAAAG

TATATGGTATACATATATATGGAAAAAAAAGTTATCCTTCGAGCCGGATTTGAACCAGCGACCTATGGATGCCTGCTGGA

TTCGATCACCTGGAAAGTGTGATCAGATCTGAAAACTTCTACAGTCCACCGCTCTACCAACTGAGCTATCGAAGGTGATA

GCAGCTCAGGTGTCAATTTAAAATTTCGCCAGCTTATCAAAATTAGTTCAAGTACTCGGCAAAAAGCAGATTCAAGTGAA

ATAATGGCAAAAAGCTTATCGCAGCATTAAAACGCAGCAACAACACCGTCACAGGTATAccccaatacgtgttgataaga

GAGAGggaagcaagtgccaaagcgcctcgagtggcgcgccggtaccGAAGTTCCTATTCCGAAGTTCCTATTCTCTAGAA

AGTATAGGAACTTCCCTAGGGCGGCCGCTAGAGGTAGTCCTTTTTTTTAGATTCAATAGCTAAGTTcctatgatttcttt

gctccaagcTCACCCGCAATATCCTTCTTTTCCGTTTCCTTGCAGATagtaaaatcgacgatcgatttccgcaccaccac

cggtttcggctgcacttcgacgtgaagagcattcccgccgacgagaagctgaaggcggcggagCTGCAGctgaCCCGGGa

cgcactcagtcaacaggtggtggccagcagatcgtcggcgaatcggacgcgctaccaggtgcttgtctacgacatcacgc

gcgtcggggtgcgtggtcagcgggagccgagctatctgctgttggacaccaagacggtccggcttaacagcacggacacg

gtgagcctcgatgtccagccggccgtggaccggtggctggcgagtccgcagcgcaactacggactgctggtggaggtgcg

gacggtccgctccctgaagccggccccacaccaccatgtacgcctgcgccgcagcgcggaCGAGGCGCACGAGCGGTGGC

AGCacaagcagccGCTCCTGTTCACCTACACGGACGacgggcggcacaaggcgcgctccattcgggacgtgtctggcgga

gagggcggtggcAAGGGCGGCCGGAACAAGCGGCAGCCGAGACGGCCTACGAGGCGCAAGAACCACGACGACtacccata

cGACGTCctgaattcGTCGAAACTGTTTTCTGCCATGAGGGACACGAATAATAAGTACGTTCTCACCCTGAACAAGTTCA

GCAAGGAAAACGAAGGCTACTATTTCTGCTCAGTCATCAGCAACTCGGTGATGTACTTCAGTTCTGTCGTGCCAGTCCTT

CAGAAAGTGAACTCTACTACTACCAAGCCAGTGCTGCGAACTCCCTCACCTGTGCACCCTACCGGGACATCTCAGCCCCA

GAGACCAGAAGATTGTCGGCCCCGTGGCTCAGTGAAGGGGACCGGATTGGACTTCGCCTGTGATATTTACATCTGGGCAC

CCTTGGCCGGAATCTGCGTGGCCCTTCTGCTGTCCTTGATCATCACTCTCATCTGCTACCACAGCCGCGGATCCATGAGT

AAAGGAGAAGAACTTTTCACTGGAGTTGTCCCAATTCTTGTTGAATTAGATGGTGATGTTAATGGGCACAAATTTTCTGT

CAGTGGAGAGGGTGAAGGTGATGCAACATACGGAAAACTTACCCTTAAATTTATTTGCACTACTGGAAAACTACCTGTTC

CATGGCCAACACTTGTCACTACTTTAACTTATGGTGTTCAATGCTTTTCAAGATACCCAGATCATATGAAACAGCATGAC

TTTTTCAAGAGTGCCATGCCCGAAGGTTATGTCCAGGAAAGAACTATATTTTTCAAAGATGACGGGAACTACAAGACACG

TGCTGAAGTCAAGTTTGAAGGTGATACCCTTGTTAATAGAATCGAGTTAAAAGGTATTGATTTTAAAGAAGATGGAAACA

TTCTTGGACACAAATTGGAATACAACTATAACTCACACAATGTATACATCATGGCAGACAAACAAAAGAATGGAATCAAA

GTTAACTTCAAAATTAGACACAACATTGAAGATGGAAGCGTTCAACTAGCAGACCATTATCAACAAAATACTCCAATTGG

CGATGGCCCTGTCCTTTTACCAGACAACCATTACCTGTCCACACAATCTGCCCTTTCGAAAGATCCCAACGAAAAGAGAG

ACCACATGGTCCTTCTTGAGTTTGTAACAGCTGCTGGGATTACACATGGCATGGATGAACTATACAAATAATCTAGTGGA

ACCTTACTTCTGTGGTGTGacataattggacaaactacctacagagatttaaagctctaaggtaaatataaaatttttaa

gtgtataatgtgttaaactactgattctaattgtttgtgtattttagatTCCAACCTATGGAACTGATGAATGGGAGCAG

TGGTGGAATGCCTTTAATGAGGAAAAcctgttttgctcagaagaaatgccatctagtgatgatgaggctactgctgactc

tcaacattctactcctccaaaaaagaagagaaaggtagaagaccccaaggactttccttcagaattgctaagttttttga

gtcatgctgtgtttagtaatagaactcttgcttgctttgctatttacaccacaaaggaaaaagctgcactgctatacaag

aaaattatggaaaaatatttgatgtatagtgccttgactagagatcataatcagccataccacatttgtagaggttttac

ttgctttaaaaaacctcccacacctccccctgaacctgaaacataaaatgaatgcaattgttgttgttaacttgtttatt

gcagcttataatggttacaaataaagcaatagcatcacaaatttcacaaataaagcatttttttcactgcattctagttg

tggtttgtccaaactcatcGGGCCC
